# Copy number variant detection using next-generation sequencing in *EYS*-associated retinitis pigmentosa

**DOI:** 10.1371/journal.pone.0305812

**Published:** 2024-06-24

**Authors:** Masakazu Hiraoka, Yusaku Urakawa, Kanako Kawai, Akiko Yoshida, Junichi Hosakawa, Masaki Takazawa, Akira Inaba, Satoshi Yokota, Yasuhiko Hirami, Masayo Takahashi, Osamu Ohara, Yasuo Kurimoto, Akiko Maeda

**Affiliations:** 1 Department of Ophthalmology, Kobe City Eye Hospital, Kobe, Japan; 2 Department of Ophthalmology, Kawasaki Medical School, Kurashiki, Okayama, Japan; 3 Department of Ophthalmology, Kobe City Medical Center General Hospital, Kobe, Japan; 4 Department of Frontier Research and Development, Laboratory of Medical Omics Research, Kazusa DNA Research Institute, Chiba, Japan; 5 Vision Care Inc., Kobe, Japan; 6 Research Organization of Science and Technology SR Center, Ritsumeikan University, Shiga, Japan; CNR, ITALY

## Abstract

Retinitis pigmentosa (RP) is the most common inherited retinal dystrophy and a major cause of blindness. RP is caused by several variants of multiple genes, and genetic diagnosis by identifying these variants is important for optimizing treatment and estimating patient prognosis. Next-generation sequencing (NGS), which is currently widely used for diagnosis, is considered useful but is known to have limitations in detecting copy number variations (CNVs). In this study, we re-evaluated CNVs in *EYS*, the main causative gene of RP, identified via NGS using multiplex ligation-dependent probe amplification (MLPA). CNVs were identified in NGS samples of eight patients. To identify potential CNVs, MLPA was also performed on samples from 42 patients who were undiagnosed by NGS but carried one of the five major pathogenic variants reported in Japanese *EYS*-RP cases. All suspected CNVs based on NGS data in the eight patients were confirmed via MLPA. CNVs were found in 2 of the 42 NGS-undiagnosed RP cases. Furthermore, results showed that 121 of the 661 patients with RP had *EYS* as the causative gene, and 8.3% (10/121 patients with *EYS*-RP) had CNVs. Although NGS using the CNV calling criteria utilized in this study failed to identify CNVs in two cases, no false-positive results were detected. Collectively, these findings suggest that NGS is useful for CNV detection during clinical diagnosis of RP.

## Introduction

Retinitis pigmentosa (RP) is the most common inherited retinal dystrophy (IRD), with an incidence of 1 in 4000 individuals, and more than 70 genes are known to cause this disease [1]. Next-generation sequencing (NGS) is widely used for genetic testing of IRD [2]. Although genetic testing using NGS has evolved over the years, only 55–60% of IRD cases are diagnosed genetically [3, 4]. Structural, splicing, and intron mutations, such as copy number variations (CNVs), are responsible for the declining diagnostic rates [4, 5].

CNVs are molecular phenomena wherein genome sequences are repeated, and the number of repeats may differ between individuals of the same species [6]. Although CNVs may not exert any eventual effect, accumulating evidence indicates that they are associated with the pathogenesis of various diseases [7, 8]. Currently, there are several tools for the identification of CNVs from NGS data; however, it remains difficult to accurately detect and interpret CNVs [9]. False-positives are often a problem while detecting CNVs using NGS; therefore, CNVs identified using NGS should be confirmed through other methods, particularly when the results are of clinical importance [10]. *EYS* is one of the main causative genes of RP worldwide and is a primary cause in the Japanese population [11–20]. CNVs in *EYS* were found to be pathogenic in approximately 15% of all families with a single heterozygous pathogenic *EYS* variant [13] and 13.2% of all Japanese patients, which suggests an important role of CNVs in *EYS-*associated RP [11]. Additionally, as the deletion of one or more exons has been reported to account for a significant portion of pathogenic variants in *USH2A* and *PRPF31*, which are also major causal genes for RP, the precise detection of CNVs is critical for genetic testing [21–23]. In this study, the CNVs identified in *EYS* using NGS were re-evaluated using multiplex ligation-dependent probe amplification (MLPA) to investigate the accuracy of CNV detection by NGS.

## Materials and methods

### Ethical guidelines

Patient recruitment was conducted between September 4, 2020 and January 31, 2023. Samples from all patients and family members were acquired according to the principles of the Declaration of Helsinki, and written informed consent was obtained to accompany the patient samples. For minors, parental consent was obtained. The Research Review Committee of Kobe City Kobe Eye Center Hospital approved the study protocol (Permit No. E19002). Genomic DNA was isolated from EDTA blood according to standard protocols. Data were obtained between February 22, 2023 and July 13, 2023. The authors did not have access to any personally identifiable information of the participants during and after data collection.

### Patients

This study included 661 patients with RP. Inclusion criteria were a clinical diagnosis of IRD and a request for genetic analysis following genetic counseling. A complete ophthalmological examination was performed for diagnosis, and RP was confirmed based on the occurrence of bilateral visual loss, night blindness, visual field constriction, narrow retinal vessels, coarse retinal pigmentation, bone spicule pigmentation, white spots, optic nerve atrophy, and macular degeneration in the fundus. Visual fields were examined using the Humphrey Field Analyzer (Carl Zeiss-Humphrey Systems, Dublin, California, USA) and Goldmann perimetry (Haag Streit, Bern, Switzerland). Electroretinograms (LE-4000; Tomey, Nagoya, Japan) were examined for attenuation and loss. Retinal pigment epithelium and photoreceptor cells were evaluated using fundus autofluorescence (Optos 200Tx; Optos, Dunfermline, Scotland) and optical coherence tomography images (Spectralis; Heidelberg Engineering, Heidelberg, Germany).

### NGS and variant analyses

Targeted NGS using a 50-gene panel was performed for the initial genetic testing (S1 Table). Targeted libraries were sequenced on an Illumina NextSeq 500 (Illumina, San Diego, CA, USA). The detected variants were interpreted based on the criteria and guidelines recommended by the American College of Medical Genetics and Genomics and the Association for Molecular Pathology [23]. The molecular diagnosis of each patient was reviewed by a multidisciplinary team that included ophthalmologists, clinical geneticists, optometrists, nurses, researchers, and genetic counselors.

### CNV detection using NGS

First, the total depths of each specimen were summed. Next, each depth was divided by the total depth at each position on each specimen. These data were calculated as means and standard deviations of depth per position for all samples. Then, outliers beyond 2 standard deviations were selected for each specimen.

### MLPA and CNV analysis

MLPA was performed using the MLPA kit P328-A3 designed for *EYS* (MRC-Holland, Amsterdam, Netherlands). The Probemix contained 55 MLPA probes with amplification products of 128–500 nt. The P328-A3 contained one probe for each exon or near-exon of the gene, except for exons 9 and 27. A probe was included for each intron, and this probe mix also included probes for introns 11 and 27 and exons 12, 17, and 28, which were normal copy number probes. Additionally, nine references were used to detect different autosomal positions. Capillary electrophoresis was performed using an ABI 3500 capillary sequencer (Applied Biosystems, Foster City, CA). Data analysis was performed using the Coffalyser.Net software (www.mrcholland.com).

## Results

**Fig 1:**
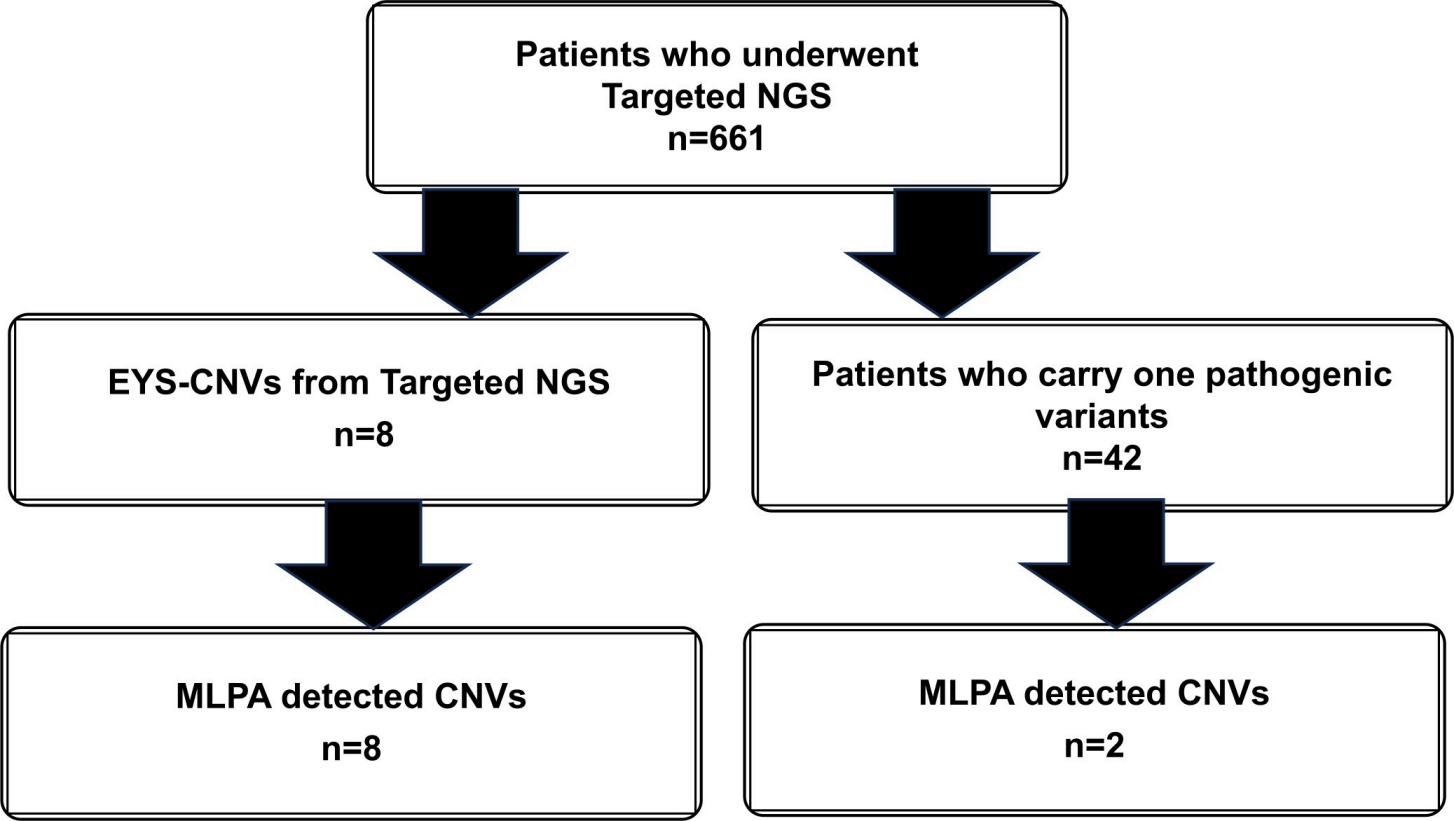
Patients selected from the NGS panel test to confirm CNVs via MLPA.

**Fig 2:**
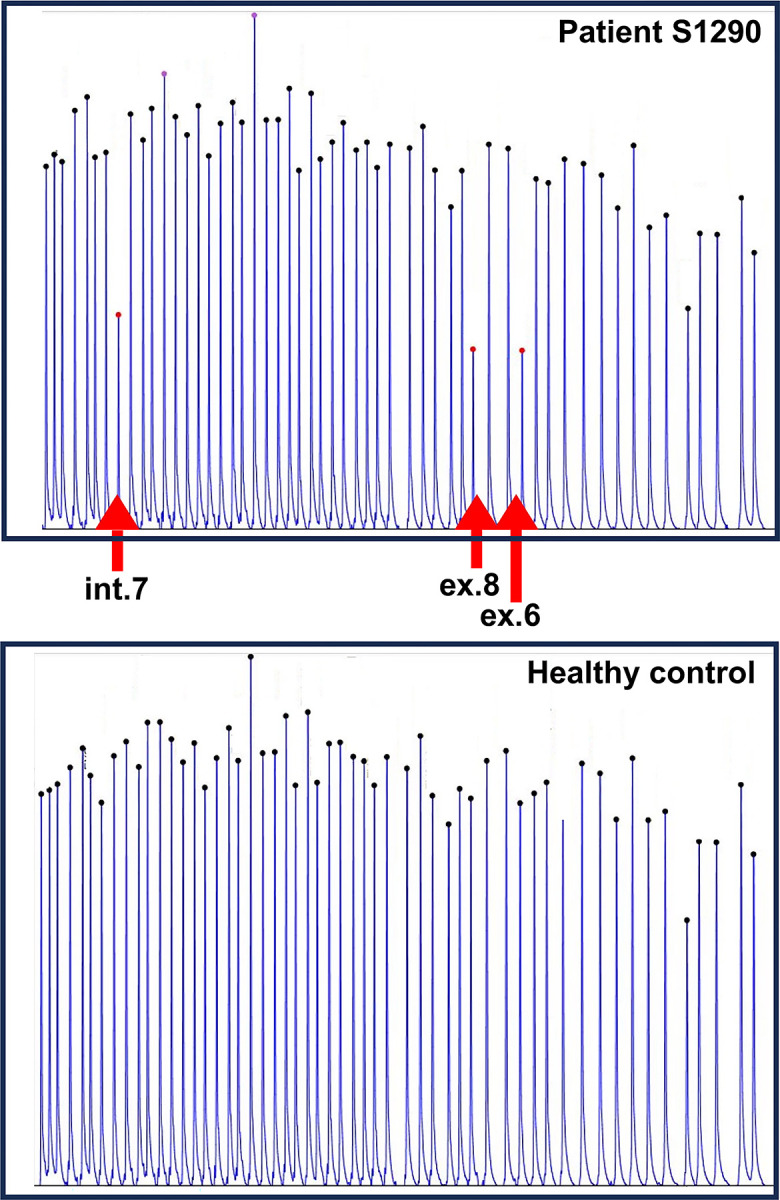
MLPA profiles of control individuals and patients with RP. A loss of signal was observed in *EYS* exons 6, 7, 8, 23, and 28 in patients with RP compared to negative controls. The absence of *EYS* peaks in these exons is indicated by *arrows*.

Targeted NGS identified 8 patients with CNVs in *EYS* and 42 undiagnosed patients carrying one of the five major *EYS* pathogenic variants (Fig 1 and Table 1). First, the CNVs detected using NGS in eight patients were further evaluated using MLPA, which is a standard method for detecting CNVs. Representative results from patient S1290 and a healthy control are shown in Fig 2. In this patient, MLPA successfully detected the deletion of intron 7 and exons 8 and 6, indicating the deletion of exons 6–8. These deletions were not detected in the healthy control. Similarly, MLPA detected the CNVs identified by NGS in seven other patients (Table 2). In all the eight patients, the CNVs identified using NGS were confirmed using the MLPA method.

**Table 1:** Five major *EYS* pathogenic variants in the Japanese population, and the number of undiagnosed patients in this study.

DNA Changes	Protein Effects	Patients (males, females)	Reference
c.2528G>A	p.(Gly843Glu)	16 (M = 10; F = 6)	[24, 25]
c.4957dupA	p.(Ser1653Lysfs*2)	14 (M = 8; F = 6)	[17–19]
c.8868C>A	p.(Tyr2956*)	8 (M = 4; F = 4)	[24, 26]
c.6557G>A	p.(Gly2186Glu)	2 (M = 0; F = 2)	[25, 26]
c.6563T>C	p.(Ile2188Thr)	2 (M = 1; F = 1)	[26]
		**Total 42**	

**Table 2:** CNVs were detected via MLPA in all eight patients predicted by NGS.

Patient ID	Exon	Nucleotide exchange	Reference
S825	6–8	Deletion	[11]
S858	6–8	Deletion	[11]
S983^§^	6–8	Deletion	[11]
S985	4–12	Deletion	This study
S1007	32–33	Duplication	This study
S1290^§^	6–8	Deletion	[11]
S1520	23–28	Deletion	This study
S1530	23–28	Deletion	This study

^§^S983 and S1290 were siblings

**Fig 3:**
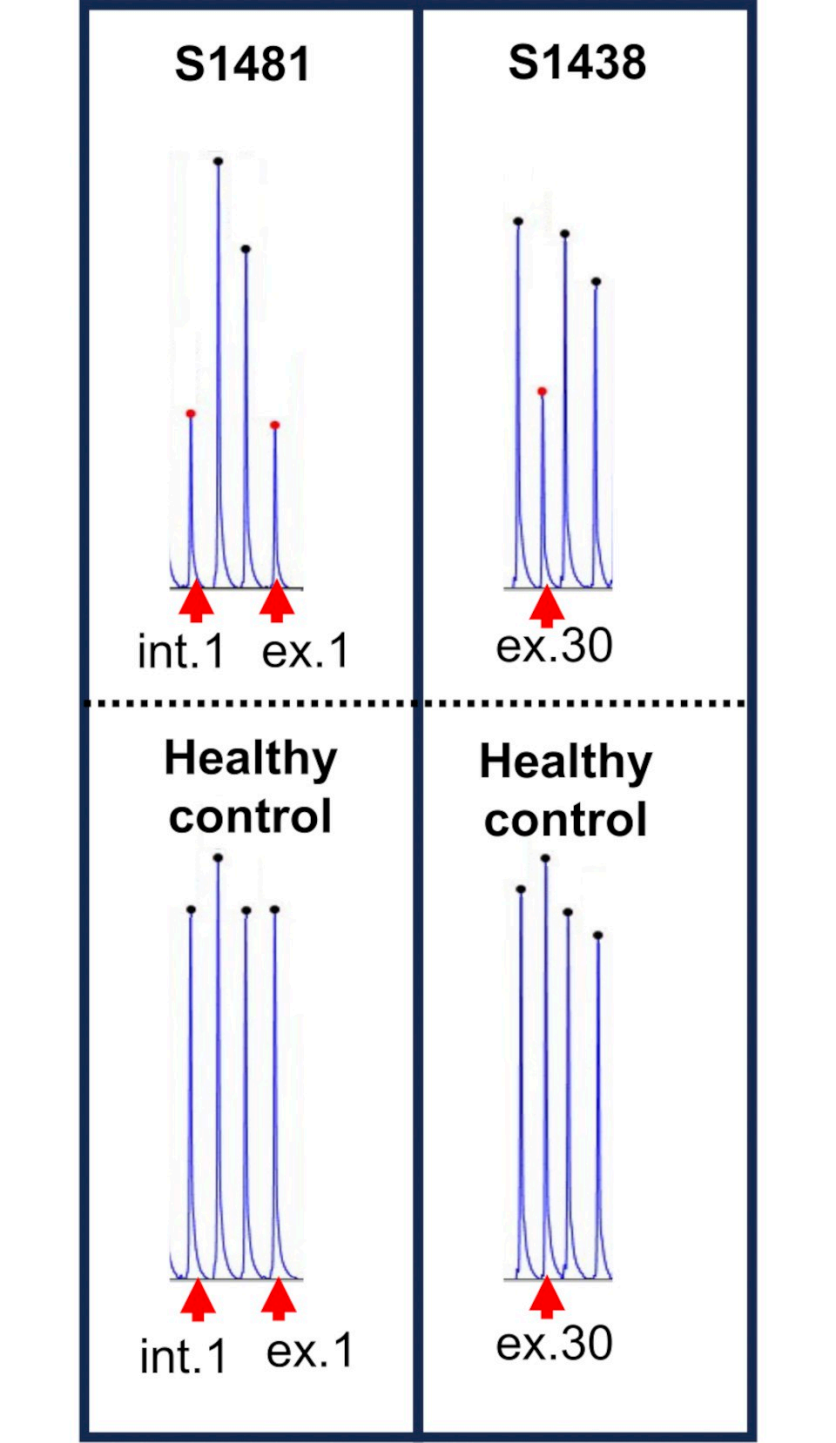
Two CNVs detected in 42 patients carrying a single heterozygous variant using MLPA. MLPA profiles of control individuals (bottom row) and patients with CNVs (top row). CNVs were identified in two cases, with deletions of exons 1 and 30 and intron 1 in *EYS*.

**Fig 4:**
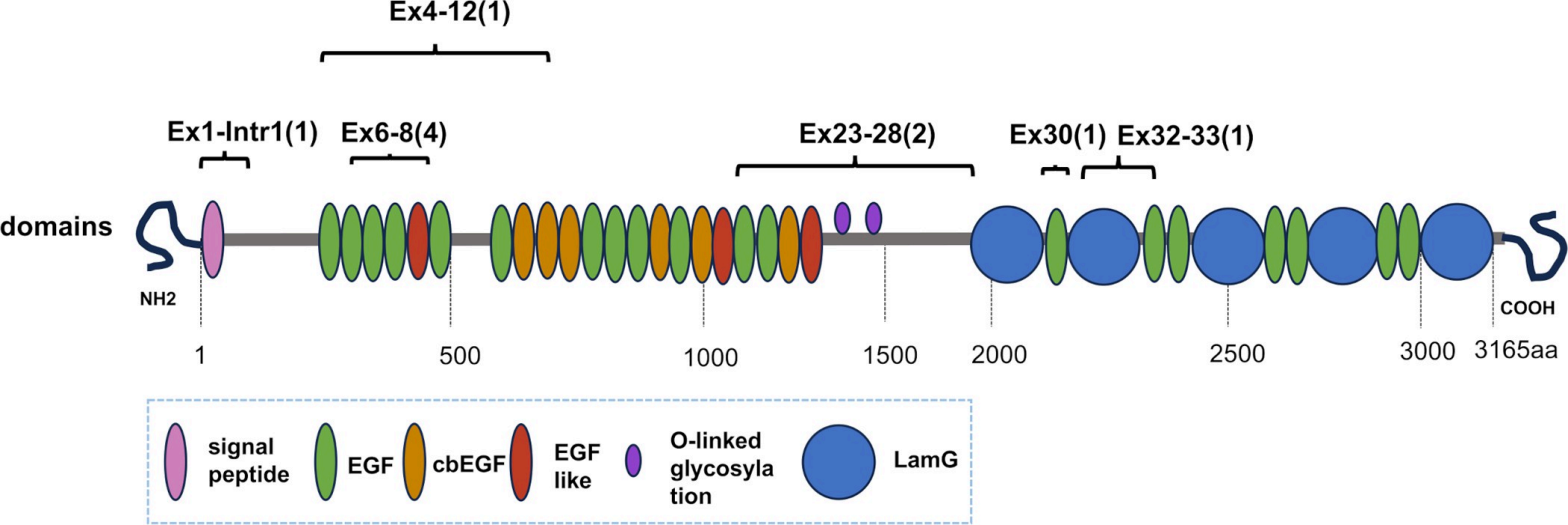
Gene structure of human EYS and predicted protein domain organization, with locations corresponding to CNVs. The number of patients identified is indicated in brackets.

Next, we performed MLPA on samples from 42 patients who carried one of the five major pathogenic variants among Japanese *EYS*-RP cases but were undiagnosed by NGS. Among these 42 patients, c.4957dupA (p.Ser1653Lysfs*2) was found in 13, c.8868C>A (p.Tyr2956*) was found in 8, c.2528G>A (p.Gly843Glu) was detected in 16, c.6557G>A (p.Gly2186Glu) was found in 2, and c.6563T>C (p.Ile2188Thr) was detected in 3 patients (Table 1). MLPA detected CNVs in 2 of the 42 (4.8%) patients—exon 1–intron 1 deletion in patient S1481 and exon 30 deletion in patient S1438 (Fig 3). Considering this result, among the 611 patients with RP, 121 had *EYS* as the causative gene and 8.3% (10/121 patients with *EYS*-RP) harbored CNVs. Novel CNVs were found in three cases—deletion of exons 23–28 and 30 and duplication of exons 32–33 (Fig 3). The locations of the CNVs found in this study are shown in Fig 4. The deletion of exons 6–8, the most frequently observed CNVs in this study, was found in four patients.

## Discussion

Several reports claim that the methods for detecting CNVs from panel-based targeted NGS data are highly sensitive, specific, and accurate [27–29]. However, detecting CNVs using panel-based targeted NGS is generally not recommended, and CNVs identified via NGS should be verified using other methods, such as PCR, allele-based comparative genomic hybridization, and MLPA [10, 14]. PCR and allele-based comparative genomic hybridization are typically employed to detect moderate deletions and duplications; however, the throughput of these methods is limited [13, 15, 30].The MLPA method used in this study allows 50 different probes to react simultaneously, and the entire reaction can be completed in a single tube. Additionally, MLPA requires only a thermal cycler and a capillary sequencer and is relatively easy and cost-effective to implement in genetic laboratories. MLPA is a type of multiplex PCR method that detects the copy number of DNA regions. Probes are designed to hybridize adjacent to the DNA sequence of interest, and after hybridization, a ligation reaction is performed to amplify the probes [31, 32]. Several studies have shown that structural changes, such as CNVs, act as pathogenic variants and that MLPA is a gold standard for detecting CNVs [21, 28].

In this study, we investigated the utility of NGS for detecting CNVs by performing MLPA to verify the CNVs detected in *EYS* through NGS. Additionally, we analyzed a single heterozygous case with only one allele of a pathogenic *EYS* variant, which is a common occurrence in Japanese patients. *EYS* is the largest gene expressed in the human eye, and its function may contribute to the maintenance of photoreceptor structure. Japanese patients with RP exhibit high detection rates (15.0–32.8%) for *EYS*, which stands as the most frequently identified causal gene [24, 25, 32]. The assessment of CNVs is important for understanding the pathogenesis of *EYS*-RP and other causal genes associated with RP. In our cohort, MLPA successfully identified 10 CNVs, including 8 CNVs identified by NGS. Notably, all the eight CNVs detected by NGS were confirmed via MLPA. These results strongly suggest that NGS is a useful tool for detecting CNVs when proper annotations are employed. Additionally, CNVs detected by NGS are likely accurate, with false-positives being of minimal concern.tingly, this study identified 2 CNVs in 42 patients who were not assessed using NGS. Although the current method underestimates CNVs, NGS can serve as a powerful clinical diagnostic tool for CNV detection.

A limitation of this study is the absence of segregation analyses, which could help in elucidating pathogenicity. Without such analyses, variants in patients cannot be confirmed as heterozygous. Moreover, a lack of detailed phenotypic data and segregation analysis may lead to a misdiagnosis of variants as false positives. Incorporating these analyses would contribute to a comprehensive diagnosis.

Techniques such as long-read sequencing, which can directly detect structural mutations, have superior detection of CNVs compared to short read sequencing [33]. Many false-positive CNVs are called from CNV detection tools [34]. However, better performance was reported by tools that analyze the variance of the depth of coverage of individual amplicons [35]. If these techniques were used, more accurate detection could have been achieved.

Collectively, we demonstrated that no false-positives were found in *EYS*-RP cases predicted to have CNVs using NGS, suggesting that NGS is a useful and complementary diagnostic tool for CNV detection in patients with IRD.

## Supporting information

S1 Fig Data analyzed by MLPA. (TIF)

S1 Table Details of genes included in the 50-gene panel. (TIF)
