# Supplementary figures and images for: Copy number variant detection using next-generation sequencing in EYS-associated retinitis pigmentosa
Source: PLoS One. 2024 Jun 24;19(6):e0305812. doi: 10.1371/journal.pone.0305812 (PMC11195993; doi:10.1371/journal.pone.0305812)

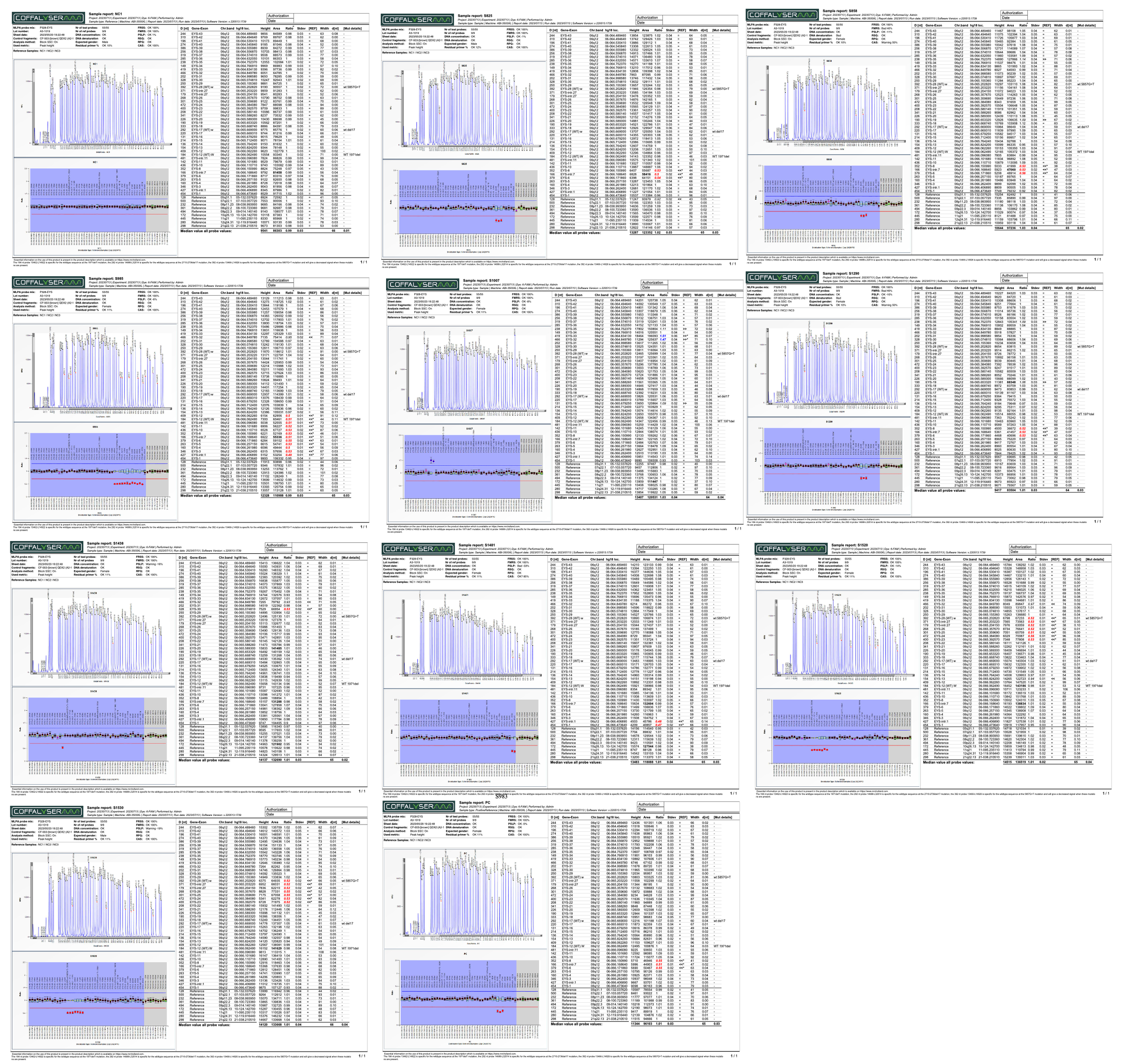

Supplement: S1 Fig — (TIF) [file pone.0305812.s001.tif]

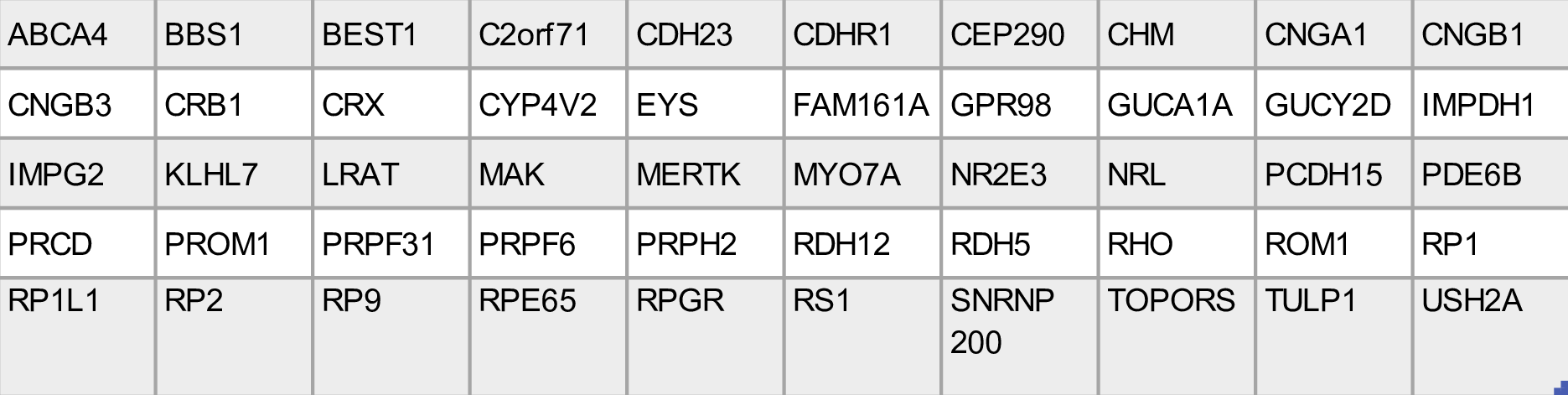

Supplement: S1 Table — (TIF) [file pone.0305812.s002.tif]
